# Racial and Ethnic Disparities in Pharmacologic and Non‐Pharmacologic Pain Management Among Older Cancer Survivors

**DOI:** 10.1002/cam4.71536

**Published:** 2026-01-23

**Authors:** Oindrila Bhattacharyya, Mohamed I. Elsaid, Brittany E. Punches, Andy Ni, Ashley S. Felix, Macarius M. Donneyong

**Affiliations:** ^1^ Comprehensive Cancer Center, College of Medicine The Ohio State University Columbus Ohio USA; ^2^ Department of Biomedical Informatics, College of Medicine The Ohio State University Wexner Medical Center Columbus Ohio USA; ^3^ Center for Biostatistics, College of Medicine The Ohio State University Columbus Ohio USA; ^4^ Department of Internal Medicine, Division of Medical Oncology, College of Medicine The Ohio State University Columbus Ohio USA; ^5^ College of Nursing The Ohio State University Columbus Ohio USA; ^6^ Department of Emergency Medicine The Ohio State University College of Medicine Columbus Ohio USA; ^7^ Division of Biostatistics The Ohio State University College of Public Health Columbus Ohio USA; ^8^ Division of Epidemiology The Ohio State University College of Public Health Columbus Ohio USA; ^9^ Division of Pharmacy Practice and Science The Ohio State University College of Pharmacy Columbus Ohio USA

## Abstract

**Introduction:**

Pain is common among cancer survivors and often managed with medication; however, racial‐ethnic disparities persist in pain treatment. Given inadequate data on pharmacologic and non‐pharmacologic pain management among minoritized cancer survivors, we investigated these patterns using the SEER‐Medicare claims linked database.

**Methods:**

An incident cancer diagnosis cohort (≥ 66 years) was created from 2007 to 2016. The primary outcome was incidence of pain treatment (pharmacologic and non‐pharmacologic) within 90 days post‐diagnosis. Racial disparities were measured as adjusted incidence ratios for treatment using Poisson regression and adjusted differences in supply days and doses using linear regression, comparing non‐Hispanic Black (nHB), Hispanic‐Latino (LatinX), Asian/Pacific Islander (API), and Others to non‐Hispanic White (nHW). Models were adjusted for demographic and clinical variables.

**Results:**

Among 300,048 survivors—nHW (72.9%), nHB (9.6%), LatinX (8.8%), API (7.3%), Others (1.4%)—nHB (aIR = 0.96, 95% CI, 0.95–0.98), API (aIR = 0.96, 95% CI, 0.95–0.98) and Others (aIR = 0.91, 95% CI, 0.87–0.94) used less pain treatment overall than nHW. Opioids (80% of treatments) were less frequently utilized by males of the Other race‐ethnicity group (aIR = 0.75, 95% CI, 0.70–0.81), compared to nHW males. For non‐opioids, nHB males (aIR: 0.84, 95% CI, 0.79–0.88) were less likely to use these therapies as compared to nHW males. Non‐pharmacological treatments were less utilized by nHB (aIR = 0.65, 95% CI, 0.62–0.69) and LatinX survivors (aIR = 0.69, 95% CI, 0.65–0.73), as compared to nHWs. Minority survivors received lower opioid doses for longer durations, but higher non‐opioid doses for shorter durations.

**Conclusion:**

There are considerable racial‐ethnic disparities in the utilization of pharmacologic and non‐pharmacologic pain treatments among cancer survivors. Future research should explore the drivers of these inequities.

## 1 Introduction

Chronic pain is common among cancer survivors, affecting nearly eight million individuals in the U.S. [1], and is linked to poor quality of life, impaired functioning, and lower overall survival [1, 2]. In cancer survivors, pain may stem from the tumor itself, diagnostic or therapeutic procedures, or treatment‐related adverse events [3] but is often underreported and undertreated among older adults [4], with the situation potentially worse for older racial‐ethnic minority groups, particularly non‐Hispanic Black (nHB) [5], and Hispanic‐Latino (LatinX) [6] individuals. This can have serious repercussions on various aspects of their lives, including biological, psychological, and social well‐being, compounding the disproportionate biopsychosocial challenges they already face [5, 7].

Pain treatment guidelines from the World Health Organization (WHO) [8], the National Comprehensive Cancer Network (NCCN) [9], and the American Society for Clinical Oncology (ASCO) [10] recommend opioids for cancer survivors who are unresponsive to non‐opioid or adjuvant analgesics. However, over the past decade, providers have grown increasingly reluctant to prescribe controlled substances due to concerns over legal scrutiny, potential prosecution [11], and the influence of the Centers for Disease Control and Prevention (CDC) guidelines on prescribing opioids for chronic pain [12], along with recent legislation aimed at curbing opioid misuse [13, 14, 15]. This climate of uncertainty, coupled with stereotypes that racial minorities are more likely to abuse pain medication [16], may worsen the disparities in pain management. Indeed, in the non‐cancer setting, it is well established that nHB and LatinX individuals are less likely to receive opioids for migraines, back pain [17], or other pain‐related diagnoses [18] than non‐Hispanic White (nHW) individuals.

Limited evidence suggests that pain management is inadequate among racial‐ethnic minority cancer survivors compared to their nHW counterparts [4, 19, 20, 21, 22]. A retrospective study of nearly 14,000 cancer survivors (≥ 65 years) in U.S. nursing homes found that nHB survivors had 1.6‐times higher odds of receiving no analgesia compared to nHW survivors [4]. Another study reported that among cancer survivors in hospice or end‐of‐life care, nHB and LatinX survivors were less likely to receive opioids but more likely to undergo urine drug screening than nHW survivors [19]. However, these findings are not generalizable to survivors outside hospice or nursing homes. Additionally, there is limited data on racial‐ethnic disparities in receipt of non‐pharmacological pain treatments, although some evidence exists in non‐cancer settings [20, 21, 22]. In this study, we examined differences in the use of any prescribed pain treatment, pharmacologic‐only (including opioid and non‐opioid analgesics) and non‐pharmacologic‐only pain treatments among minoritized older cancer survivors—including non‐Hispanic Black (nHB), Hispanic‐Latino (LatinX), Asian/Pacific Islander (API), and Others—relative to non‐Hispanic White (nHW) survivors.

## 2 Methods

We conducted a retrospective cohort study using 2007–2016 SEER‐Medicare data to examine racial‐ethnic disparities in pain management among cancer survivors aged ≥ 66 years. This study was approved by The Ohio State University IRB.

### 2.1 Data Sources and Study Population

The SEER registry is a large population‐based database collecting clinical, demographic, and cause‐of‐death data for newly diagnosed cancer survivors, covering approximately 28% of the U.S. population. The SEER‐Medicare database integrates cancer registry data with administrative Medicare claims from the Centers for Medicare and Medicaid Services (CMS), covering approximately 93% of SEER participants [23]. It includes data from Medicare Part D and Standard Analytic Files (SAF), offering comprehensive information on healthcare access and utilization, hospitalization, and other medical conditions.

### 2.2 Study Design

We created a cohort of individuals aged ≥ 66 years with incident diagnoses of cancer types known for racial‐ethnic disparities in survivorship (head and neck, breast, lung, colorectal, prostate, and uterine cancers). Cases were identified using ICD‐9/10 codes [24] from Medicare claims (Table S1) instead of SEER, as the Outpatient SAF and Part D prescription data are linked to claims‐based diagnoses. To ensure completeness, we also included cases recorded within six months of SEER enrollment, since approximately 10% of cases are reported during this period [25, 26] despite SEER and Medicare claims showing high concordance for diagnosis month (> 72.2%).

**FIGURE 1:**
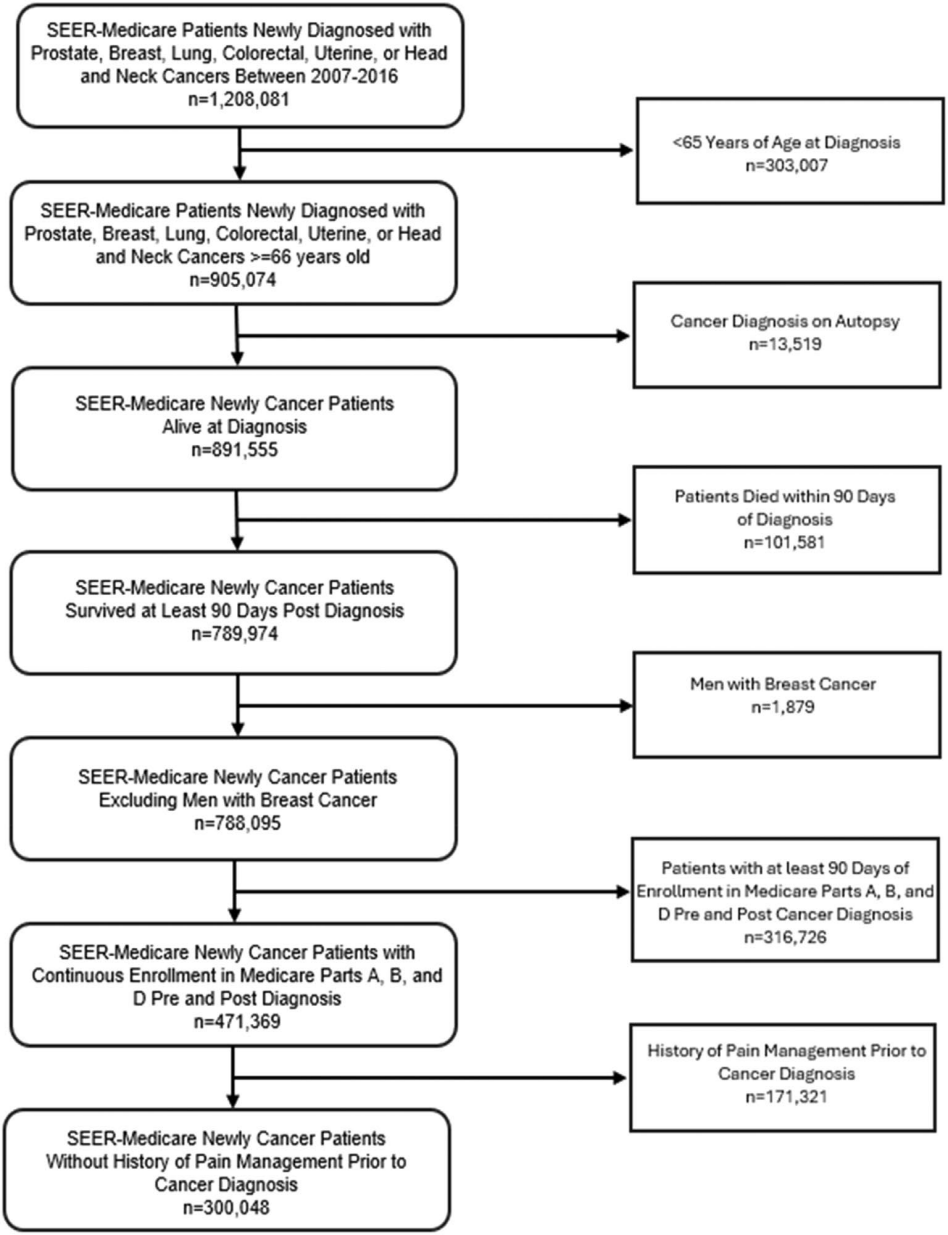
Sample derivation.

Cohort entry was the date of cancer diagnosis, with 365‐day follow‐up for incident pain treatment (defined as first receipt with no prior history). We applied a 90‐day lookback window prior to the cancer diagnosis date using Medicare Parts A, B, and D to capture any pain medication use during the three months leading up to diagnosis. Participants were required to have continuous Medicare Part D coverage (no > 30‐day gap) before and after the diagnosis. We excluded patients diagnosed at autopsy, those who died within 90 days post‐cohort entry date (since the receipt of pain therapies was evaluated within this period), men with breast cancer (due to rarity across racial‐ethnic groups) [27], and those with a history of receiving prior pain therapies (Figure 1).

### 2.3 Outcomes

The primary outcome variables were measured within the first 90 days post index date (i.e., cancer diagnosis date). These included: the receipt of any pharmacologic therapies (i.e., opioids and non‐opioid analgesics), receipt of non‐pharmacologic therapies, or the composite of both pharmacologic and non‐pharmacologic pain therapies.

Receipt of pharmacologic therapies: Pharmacologic therapies were identified via Part D claims using National Drug Codes (NDC) for opioids and non‐opioid analgesics (acetaminophen and nonsteroidal anti‐inflammatory drugs [NSAIDs], gabapentin and pregabalin, venlafaxine and duloxetine, tricyclic antidepressants [TCAs], corticosteroids, bisphosphonates and denosumab, ketamine). We defined three measures of pharmacologic treatment: incidence [28], duration [29], and dose of analgesic [30] use. For incidence, the first record of a filled prescription of any analgesic was considered an ‘incident’ if no prior analgesic fills were recorded within 180 days before that first fill date. Duration (supply days) was defined as the number of consecutive days covered by prescriptions, allowing for gaps of no more than 14 days between successive fills, until the end of the specific follow‐up period. Dose was measured as the daily defined dose (DDD) [30] in Morphine Milligram Equivalent (MME) as per the CDC guidelines [31].

Receipt of non‐pharmacologic therapies: We defined non‐pharmacologic pain therapies based on records of the receipt of—including physical medicine and rehabilitation services, integrative therapies (massage and acupuncture), interventional therapies (nerve blocks), psychological approaches (cognitive behavioral therapy), and neurostimulatory therapies. All these procedures and services were identified from Medicare claims using the respective appropriate Current Procedure Terminology (CPT) codes [32] (Table S2). The first recorded claim for any such service during the follow‐up period was considered an incident non‐pharmacologic therapy.

### 2.4 Covariates

All prespecified covariates were measured during the 180‐day period prior to the index date (inclusive). The full list of covariates is listed in Table 1. We extracted demographic and clinical data from SEER records and/or Medicare claims, guided by the literature on predictors of pain treatment receipt [35]. Demographic data included race‐ethnicity, age at diagnosis, and gender. Race‐ethnicity, the primary dependent variable, was defined from two fields—race and ethnicity (Spanish‐Hispanic‐Latino vs. not). Based on these two fields, patients were grouped into one of the following race‐ethnicity groups: nHW, nHB, LatinX, API, or Others (which included American Indian/Alaska Native, Unknown race). Clinical status included the primary cancer site, year of initial diagnosis, cancer stage (0 to IV, unknown stage), cancer treatments (radiation, chemotherapy), Charlson Comorbidity Index (0, 1 to 2, ≥ 3), and types of comorbidities (cardiovascular diseases, dementia, paralysis, diabetes with/without complications, renal and liver diseases, peptic ulcer disease, rheumatologic disease, AIDS).

**TABLE 1:** Characteristics of cancer survivors by race‐ethnicity.

Characteristics^a^	All	nHW	nHB	LatinX	API	Others	*p* ^b^
	*n* = 300,048	*n* = 218,721	*n* = 28,701	*n* = 26,427	*n* = 21,985	*n* = 4214	
Age, years							< 0.001
Mean (SD)	75.5 (7.0)	75.6 (7.0)	74.5 (6.8)	75.0 (6.7)	75.8 (7.0)	74.1 (6.3)	
Median (25th, 75th)	74.0 (70.0, 80.0)	74.0 (70.0, 80.0)	73.0 (69.0, 79.0)	74.0 (69.0, 79.0)	75.0 (70.0, 81.0)	73.0 (69.0, 78.0)	
Age, years, *n* (%)							< 0.001
66–69	71,646 (23.9)	50,943 (23.3)	7908 (27.6)	6660 (25.2)	4940 (22.5)	1195 (28.4)	
70–74	82,830 (27.6)	59,693 (27.3)	8536 (29.7)	7435 (28.1)	5836 (26.6)	1330 (31.6)	
75–79	63,649 (21.2)	46,103 (21.1)	5921 (20.6)	5851 (22.1)	4899 (22.3)	875 (20.8)	
80–84	44,885 (15.0)	33,670 (15.4)	3539 (12.3)	3741 (14.2)	3436 (15.6)	499 (11.8)	
85+	37,038 (12.3)	28,312 (12.9)	2797 (9.8)	2740 (10.4)	2874 (13.1)	315 (7.5)	
Gender, *n* (%)							< 0.001
Male	146,694 (48.9)	103,935 (47.5)	14,290 (49.8)	14,298 (54.1)	11,059 (50.3)	3112 (73.9)	
Female	153,354 (51.1)	114,786 (52.5)	14,411 (50.2)	12,129 (45.9)	10,926 (49.7)	1102 (26.2)	
Year of diagnosis, *n* (%)							< 0.001
2007	25,050 (8.4)	18,397 (8.4)	2468 (8.6)	2187 (8.3)	1745 (7.9)	253 (6.0)	
2008	34,225 (11.4)	25,033 (11.5)	3301 (11.5)	3107 (11.8)	2413 (11.0)	371 (8.8)	
2009	34,895 (11.6)	25,501 (11.7)	3281 (11.4)	3157 (12)	2554 (11.6)	402 (9.5)	
2010	34,084 (11.4)	24,693 (11.3)	3223 (11.2)	3126 (11.8)	2588 (11.8)	454 (10.8)	
2011	33,552 (11.2)	24,260 (11.1)	3198 (11.1)	3084 (11.7)	2550 (11.6)	460 (10.9)	
2012	33,465 (11.2)	24,328 (11.1)	3148 (11.0)	2946 (11.2)	2559 (11.6)	484 (11.5)	
2013	35,289 (11.8)	25,715 (11.8)	3400 (11.9)	3100 (11.7)	2560 (11.6)	514 (12.2)	
2014	34,843 (11.6)	25,472 (11.7)	3347 (11.7)	2898 (11.0)	2550 (11.6)	576 (13.7)	
2015	34,645 (11.6)	25,322 (11.6)	3335 (11.6)	2822 (10.7)	2466 (11.2)	700 (16.6)	
Type of cancer, *n* (%)							< 0.001
Prostate	85,020 (28.3)	58,914 (26.9)	9031 (31.5)	8987 (34.0)	5399 (24.6)	2689 (63.8)	
Breast	77,385 (25.8)	58,214 (26.6)	7074 (24.7)	6187 (23.4)	5293 (24.1)	617 (14.6)	
Lung	56,929 (19.0)	43,459 (19.9)	5089 (17.7)	3639 (13.8)	4488 (20.4)	254 (6.0)	
Colorectal	55,927 (18.6)	39,468 (18.0)	5376 (18.7)	5526 (20.9)	5141 (23.4)	416 (9.9)	
Uterine	14,678 (4.9)	10,856 (5.0)	1524 (5.3)	1303 (4.9)	880 (4.0)	115 (2.7)	
Head and neck	10,109 (3.4)	7810 (3.6)	607 (2.1)	785 (3.0)	784 (3.6)	123 (2.9)	
Cancer stage^c^, *n* (%)							< 0.001
0	18,348 (6.1)	13,435 (6.1)	1824 (6.4)	1460 (5.5)	1437 (6.5)	192 (4.6)	
I	72,131 (24.0)	56,003 (25.6)	5330 (18.6)	5326 (20.2)	4969 (22.6)	503 (11.9)	
II	100,218 (33.4)	71,599 (32.7)	10,514 (36.6)	9708 (36.7)	6642 (30.2)	1755 (41.7)	
III	40,853 (13.6)	29,484 (13.5)	4052 (14.1)	3666 (13.9)	3436 (15.6)	215 (5.1)	
IV	38,065 (12.7)	27,369 (12.5)	4187 (14.6)	3151 (11.9)	3175 (14.4)	183 (4.3)	
Unknown	30,433 (10.1)	20,831 (9.5)	2794 (9.7)	3116 (11.8)	2326 (10.6)	1366 (32.4)	
Charlson Comorbidity Index							< 0.001
0	164,203 (54.7)	121,099 (55.4)	13,823 (48.2)	14,886 (56.3)	11,848 (53.9)	2547 (60.4)	
1–2	101,980 (34.0)	74,105 (33.9)	10,328 (36.0)	8593 (32.5)	7590 (34.5)	1364 (32.4)	
3+	33,865 (11.3)	23,517 (10.8)	4550 (15.9)	2948 (11.2)	2547 (11.6)	303 (7.2)	
Conditions, *n* (%)							
Acute myocardial infarction	3781 (1.3)	2746 (1.3)	422 (1.5)	297 (1.1)	283 (1.3)	33 (0.8)	< 0.001
History of myocardial infarction	8011 (2.7)	6270 (2.9)	729 (2.5)	573 (2.2)	365 (1.7)	74 (1.8)	< 0.001
Congestive heart failure	24,732 (8.2)	18,004 (8.2)	3207 (11.2)	1901 (7.2)	1376 (6.3)	244 (5.8)	
Peripheral vascular disease	29,950 (10.0)	22,547 (10.3)	3219 (11.2)	2229 (8.4)	1676 (7.6)	279 (6.6)	< 0.001
Cerebrovascular disease	23,110 (7.7)	17,166 (7.9)	2595 (9)	1637 (6.2)	1455 (6.6)	257 (6.1)	
Chronic obstructive pulmonary disease	52,567 (17.5)	40,602 (18.6)	4996 (17.4)	3379 (12.8)	3161 (14.4)	429 (10.2)	< 0.001
Dementia	5361 (1.8)	3630 (1.7)	884 (3.1)	467 (1.8)	342 (1.6)	38 (0.9)	< 0.001
Hemiplegia or paraplegia	1695 (0.6)	1091 (0.5)	303 (1.1)	165 (0.6)	123 (0.6)	13 (0.3)	< 0.001
Diabetes	59,559 (19.9)	38,796 (17.7)	7792 (27.2)	6569 (24.9)	5529 (25.2)	873 (20.7)	< 0.001
Diabetes with complications	13,557 (4.5)	8387 (3.8)	2026 (7.1)	1701 (6.4)	1261 (5.7)	182 (4.3)	< 0.001
Moderate–severe renal disease	22,361 (7.5)	14,802 (6.8)	3446 (12)	1936 (7.3)	1917 (8.7)	260 (6.2)	< 0.001
Mild liver disease	11,461 (3.8)	7749 (3.5)	1155 (4.0)	1094 (4.1)	1347 (6.1)	116 (2.8)	< 0.001
Peptic ulcer disease	3889 (1.3)	2485 (1.1)	455 (1.6)	395 (1.5)	518 (2.4)	36 (0.9)	< 0.001
Rheumatologic disease	4915 (1.6)	3687 (1.7)	457 (1.6)	432 (1.6)	282 (1.3)	57 (1.4)	< 0.001
Treatment type, *n* (%)							
Radiation therapy^d^	104,835 (34.9)	78,722 (36.0)	10,034 (35.0)	8237 (31.2)	6961 (31.7)	881 (20.9)	< 0.001
Chemotherapy^d^	13,364 (4.5)	11,272 (5.2)	615 (2.1)	715 (2.7)	626 (2.9)	136 (3.2)	< 0.001
Surgical therapy	187,695 (62.6)	140,834 (64.4)	15,665 (54.6)	16,190 (61.3)	13,771 (62.6)	1235 (29.3)	< 0.001

^a^ We did not report the distribution of AIDS and moderate–severe liver disease due to cell sizes with fewer than 11 individuals. Per CMS data suppression policies, cell sizes with fewer than 11 individuals are not reported to protect patient confidentiality [33].

^b^ The associations between each characteristic and racial‐ethnic minority groups were assessed using Chi‐square tests for categorical and binary variables, and ANOVA for continuous variables.

^c^ Including survivors with not applicable stage and stage occult.

^d^ We have read and understand the limitations of the SEER Radiation and Chemotherapy data in terms of data completeness and have noted them in our analyses. We acknowledge that the SEER Program has advised us that there are substantive concerns about using these data to address certain research questions, and recognize that findings from such analyses may be inaccurate or misleading [34].

### 2.5 Statistical Analysis

We calculated crude incidence rates for the receipt of prescribed pain treatment within 90 days of diagnosis, stratified by race‐ethnicity and gender (Table S3). These rates were calculated by dividing the number of survivors with at least one documented prescription for any pain treatment by the total number of survivors eligible to receive such prescriptions during the same period. We used Poisson regression to estimate incidence rates of pain treatment receipt among racial‐ethnic minority groups, compared to nHW survivors, within 90 days post‐diagnosis. Mean daily doses (in MME/day) of the first filled prescription were calculated separately for opioids and non‐opioid analgesics. To estimate adjusted differences in analgesic doses and supply durations across racial‐ethnic groups, we used multivariate linear regression models (see Tables S4 and S5 for crude rates). All models were adjusted for age and year of diagnosis, cancer type, stage, treatment type, Charlson Comorbidity Index, and comorbidity types. Additionally, as a robustness check, we conducted sensitivity analysis to assess disparities by cancer stage and type. All analyses were performed using SAS version 9.4 (SAS Institute Inc.), with statistical significance set at *p* < 0.05.

## 3 Results

We identified 300,048 cancer survivors from the SEER‐Medicare dataset. The cohort was predominantly female (153,354, 51.1%) with a median age of 74 years (IQR 70–80) and most diagnosed at stages II‐IV (179,136, 59.7%). The majority were nHW (218,721, 72.9%), with similar representation among nHB (28,701, 9.6%), LatinX (26,427, 8.8%), and API (21,985, 7.3%) groups. nHB survivors had the highest comorbidity burden, with 36% having one or two additional conditions (*p* < 0.001). Cardiovascular diseases, diabetes alone, and diabetes with renal diseases were notably more common in this group (*p* < 0.001). Use of radiation, chemotherapy, and surgery was highest among nHW survivors (*p* < 0.001) (Table 1).

### 3.1 Any Pain Management

**FIGURE 2:**
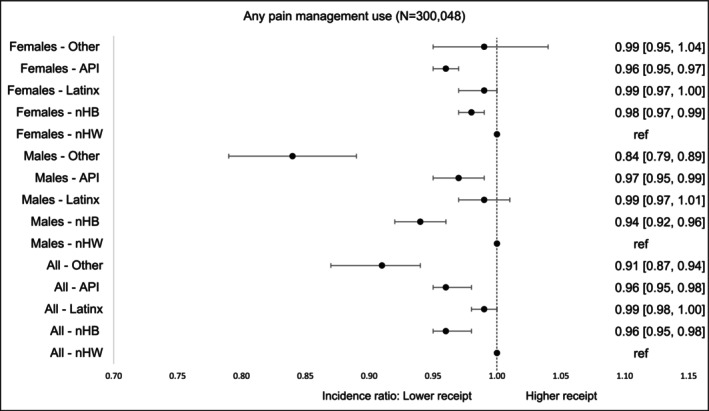
Incidence of *any pain management* by race‐ethnicity and gender within first 90 days of diagnosis. The models were adjusted for age at cancer diagnosis, year of diagnosis, type of cancer, cancer stage at diagnosis, radiation therapy, surgical therapy, chemotherapy, Charlson Comorbidity Index, type of comorbidities.

Compared to nHW survivors, racial/ethnic minority groups had slightly but significantly lower overall utilization of any prescribed pain treatments (nHB and API: aIR = 0.96, 95% CI, 0.95–0.98; Others: aIR = 0.91, 95% CI, 0.87–0.94). When stratified by gender, nHB and Other males had significantly lower utilization, with reductions of 6% and 16%, respectively. Among females, utilization was also modestly lower—2% lower for nHB survivors and 4% lower for API survivors, relative to their nHW counterparts (Figure 2).

### 3.2 Pharmacologic‐Only Pain Management

**FIGURE 3:**
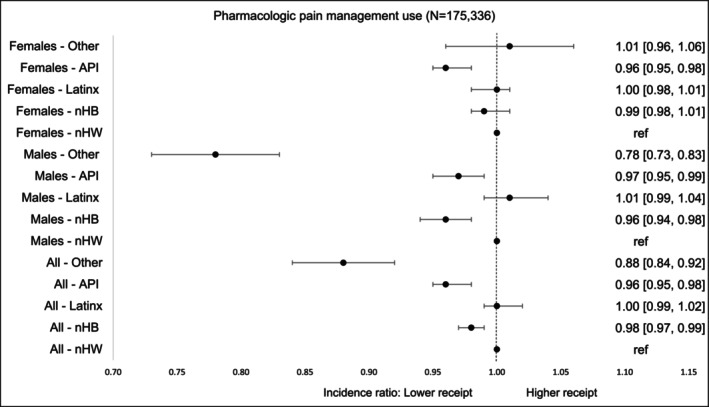
Incidence of *pharmacologic‐only pain management* by race‐ethnicity and gender within first 90 days of diagnosis. Pharmacologic pain management includes use of either opioid or non‐opioid analgesics. The models were adjusted for age at cancer diagnosis, year of diagnosis, type of cancer, cancer stage at diagnosis, radiation therapy, surgical therapy, chemotherapy, Charlson Comorbidity Index, type of comorbidities.

Pharmacologic pain treatment was less frequently utilized by racial‐ethnic minority survivors, particularly nHB (aIR = 0.98, 95% CI, 0.97–0.99), API (aIR = 0.96, 95% CI, 0.95–0.98), and Others (aIR = 0.88, 95% CI, 0.84–0.92), compared to nHW survivors. LatinX survivors showed no significant difference. This pattern persisted among minority males compared to their nHW counterparts (Figure 3).

#### 3.2.1 Opioid Analgesics

Among survivors prescribed pharmacologic analgesics (175,336, 58.4%), nearly 80% (139,598) received opioids. Opioid utilization was significantly lower among Other males (25% lower), API males (5% lower), and API females (5% lower) compared with their nHW counterparts (Figure 4A).

**FIGURE 4A:**
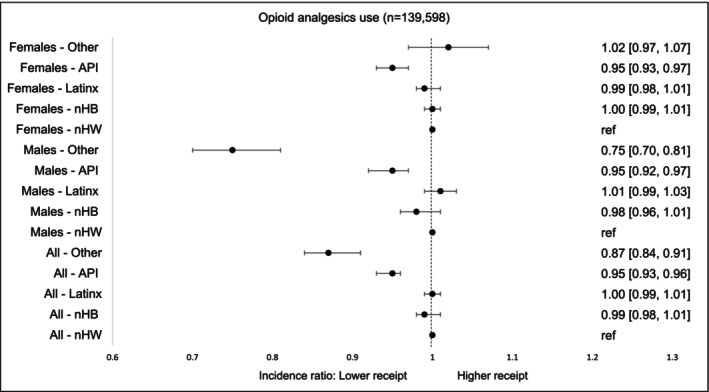
Incidence of opioid analgesics by race‐ethnicity and gender within first 90 days of diagnosis. The models were adjusted for age at cancer diagnosis, year of diagnosis, type of cancer, cancer stage at diagnosis, radiation therapy, surgical therapy, chemotherapy, Charlson Comorbidity Index, type of comorbidities.

Mean daily opioid dose was highest in nHW (46.8 MME/d, 95% CI, 46.7–47.0) and lowest in API survivors (40.7 MME/d, 95% CI, 40.2–41.2), with similar patterns across genders. Multivariate regression confirmed that all minority groups received lower doses than nHW survivors, with API survivors receiving the largest reduction (−6.1 MME/d, 95% CI, −6.6 to −5.5) (Figure 4B).

**FIGURE 4B:**
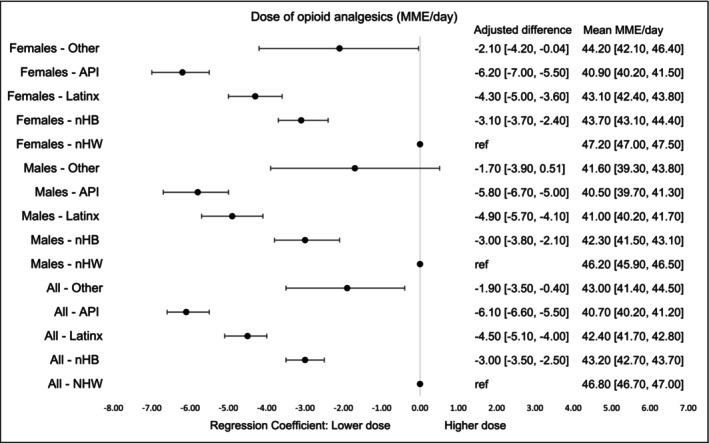
Dose of opioid analgesics (MME/d) by race‐ethnicity and gender within first 90 days of diagnosis. Dose was measured as first opioid dose filled. The models were adjusted for age at cancer diagnosis, year of diagnosis, type of cancer, cancer stage at diagnosis, radiation therapy, surgical therapy, chemotherapy, Charlson Comorbidity Index, type of comorbidities. MME = morphine milligram equivalents per day of the first prescription filled.

Opioid supply duration varied notably by race‐ethnicity. nHB survivors had the longest average duration (7.4 days, 95% CI, 7.3–7.5), with the highest levels observed among nHB and LatinX males (7.8 days, 95% CI, 7.6–8.0) compared to nHW males (7.0 days, 95% CI, 7.0–7.1). Adjusted analyses showed mean supply days were significantly higher for nHB (0.6 days, 95% CI, 0.48–0.72), LatinX (0.8 days, 95% CI, 0.66–0.91), and API (0.2 days, 95% CI, 0.12–0.36) survivors compared to their nHW counterparts. These differences were consistent across genders (Figure 4C).

**FIGURE 4C:**
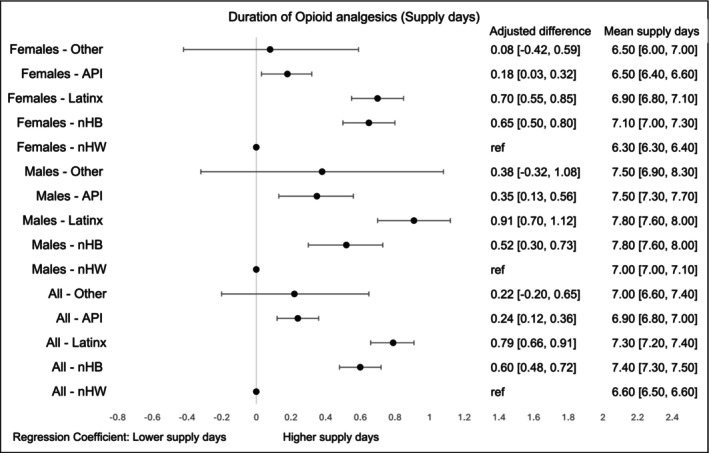
Supply days of opioid analgesics by race‐ethnicity and gender within first 90 days of diagnosis. The models were adjusted for age at cancer diagnosis, year of diagnosis, type of cancer, cancer stage at diagnosis, radiation therapy, surgical therapy, chemotherapy, Charlson Comorbidity Index, type of comorbidities.

#### 3.2.2 Non‐Opioid Analgesics

Non‐opioid analgesic use was 16% lower among nHB males (aIR: 0.84, 95% CI, 0.79–0.88) compared to nHW males. Among females, all racial‐ethnic groups—nHB, API, and Others—had significantly lower utilization than nHW survivors, with the exception of LatinX, where no significant difference was observed (Figure 5A).

**FIGURE 5A:**
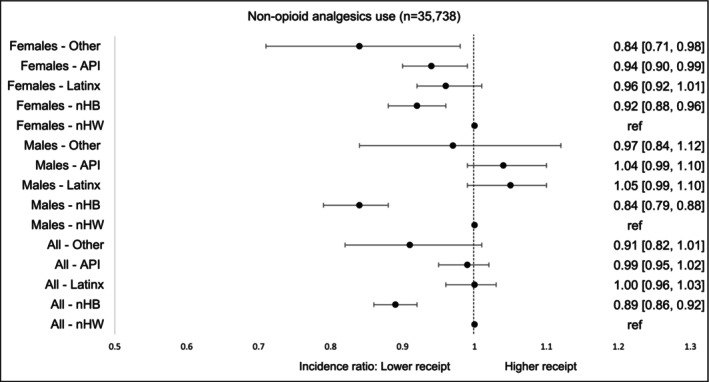
Incidence of non‐opioid analgesics by race‐ethnicity and gender within first 90 days of diagnosis. The models were adjusted for age at cancer diagnosis, year of diagnosis, type of cancer, cancer stage at diagnosis, radiation therapy, surgical therapy, chemotherapy, Charlson Comorbidity Index, type of comorbidities.

The highest mean doses were observed among survivors in the Others group (327.1 mg, 95% CI, 250.7–403.5), while API survivors had the lowest dose (178.8 mg, 95% CI, 161.0–196.5), a pattern consistent across genders. Regression estimates indicated higher doses for nHB (53.0 mg, 95% CI, 31.2–74.8) and LatinX (62.1 mg, 95% CI, 38.9–85.3) survivors overall. By gender, nHB and LatinX males had higher doses, while the largest increases among females were observed in Others, LatinX, and nHB survivors (Figure 5B).

**FIGURE 5B:**
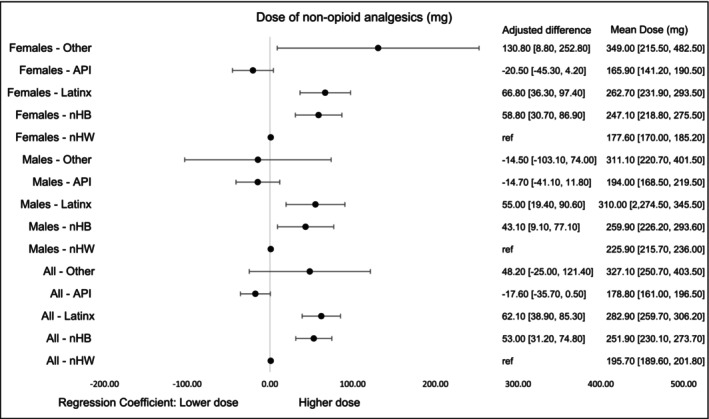
Dose of non‐opioid analgesics (mg) by race‐ethnicity and gender within first 90 days of diagnosis. Dose was measured as first opioid dose filled. The models were adjusted for age at cancer diagnosis, year of diagnosis, type of cancer, cancer stage at diagnosis, radiation therapy, surgical therapy, chemotherapy, Charlson Comorbidity Index, type of comorbidities.

Non‐opioid supply days were longest for Others (24.3 days, 95% CI, 21.4–27.2) and LatinX (22.5 days, 95% CI, 21.6–23.4), and shortest for nHB (19.1 days, 95% CI, 18.4–19.9) and API (20.7 days, 95% CI, 19.9–21.6). Adjusted mean supply days were significantly lower for nHB (−2.03, 95% CI, −2.79 to −1.27), especially among females (Figure 5C).

**FIGURE 5C:**
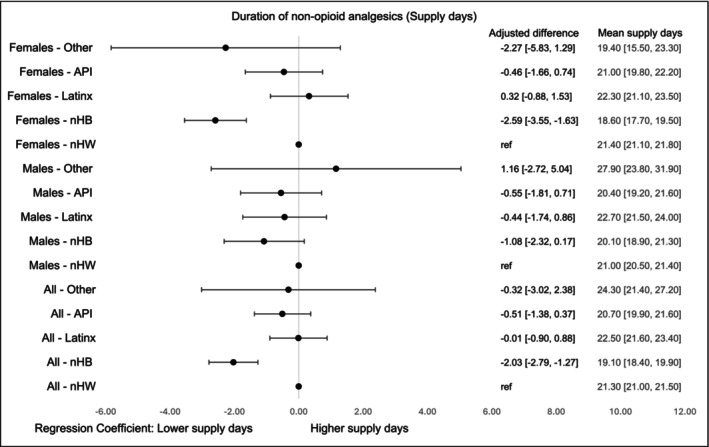
Supply days of non‐opioid analgesics by race‐ethnicity and gender within first 90 days of diagnosis. The models were adjusted for age at cancer diagnosis, year of diagnosis, type of cancer, cancer stage at diagnosis, radiation therapy, surgical therapy, chemotherapy, Charlson Comorbidity Index, type of comorbidities.

### 3.3 Non‐Pharmacologic‐Only Pain Management

**FIGURE 6:**
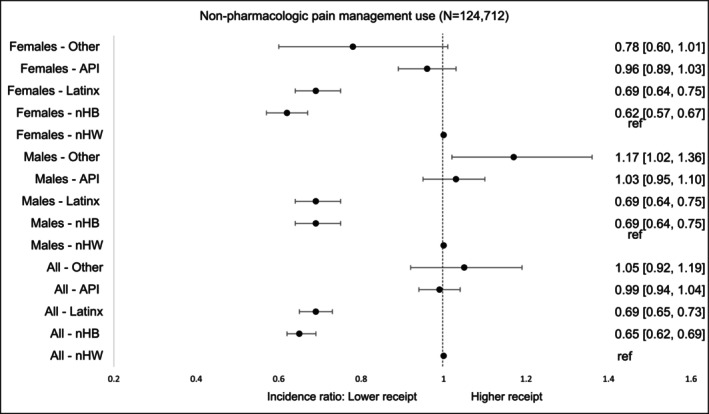
Incidence of *non‐pharmacologic‐only pain management* by race‐ethnicity and gender within first 90 days of diagnosis. The models were adjusted for age at cancer diagnosis, year of diagnosis, type of cancer, cancer stage at diagnosis, radiation therapy, surgical therapy, chemotherapy, Charlson Comorbidity Index, type of comorbidities.

Racial‐ethnic disparities were also evident in non‐pharmacologic pain treatment. nHB survivors had the lowest utilization, with rates 35% lower than those of nHW survivors (aIR = 0.65, 95% CI, 0.62–0.69). LatinX survivors also demonstrated significantly lower utilization, with rates 31% lower than nHW survivors (aIR = 0.69, 95% CI, 0.65–0.73). When stratified by gender, the disparity among nHB survivors corresponded to a 31% reduction for males and a 38% reduction for females in utilization of non‐pharmacologic pain treatment relative to their nHW counterparts. LatinX males and females each showed a 31% reduction in utilization. In contrast, males in the Other racial‐ethnic group exhibited higher utilization of non‐pharmacologic treatment, with rates 17% greater than those of nHW males (Figure 6).

### 3.4 Sensitivity Analysis

Robustness checks confirmed significant disparities among nHB and API survivors in early‐stage (I–III) cancers, particularly in prostate and breast cancer. Disparities among API survivors were limited to early stages (Tables S6 and S7).

## 4 Discussion

In this retrospective cohort study using the nationally representative SEER‐Medicare database, we identified significant racial‐ethnic disparities in the use of prescribed pain treatment among older cancer survivors. Across all groups, pharmacologic treatments were more commonly prescribed than non‐pharmacologic treatments. Racial‐ethnic minority groups, particularly nHB and API survivors, had lower overall utilization of any pain treatment compared to nHW survivors. Opioids were the most commonly used pharmacologic treatment, but utilization was significantly lower among males in the Other and API groups, as well as among API females. Non‐opioid analgesics were also less frequently utilized by nHB males and most minority females. Disparities extended to non‐pharmacologic treatments as well, with nHB and LatinX survivors showing lower utilization of prescribed treatment than nHW survivors. Among those receiving opioids, minority groups had lower average daily doses, especially API survivors, although supply durations were generally longer for all groups except Others. In contrast, non‐opioid doses were highest among LatinX survivors, while nHB survivors had shorter supply durations.

Within pharmacologic treatment, while opioids remain the most commonly used approach for cancer pain management, as evidenced in existing literature [36, 37, 38], our findings show significant variations in utilization across racial‐ethnic groups. Prior studies report mixed results [39, 40, 41, 42, 43, 44, 45, 46, 47]; some show lower use of opioids among API survivors nearing end‐of‐life [45], while others find higher odds of receiving an opioid prescription among opioid‐naive API survivors and lower odds among nHB survivors [44]. Evidence for Black cancer survivors has also been inconsistent, with some studies reporting higher short‐term opioid prescribing [40], while others found no differences compared with Whites survivors [41]. These inconsistencies likely stem from differences in study populations, research settings, and analytic approaches. Given the persistent underutilization of pain management treatments among racial‐ethnic minority cancer survivors, our findings highlight the need for targeted interventions, such as greater integration of non‐opioid options into treatment plans or improved access to referrals and counseling services for pain management. Our study is the first to document racial‐ethnic disparities in the use of prescribed non‐opioid analgesics among cancer survivors. While non‐opioids are commonly recommended for mild pain or as adjuncts to opioids [48], research on their use in cancer populations is limited, with existing studies focusing on chronic non‐cancer pain management [49] in emergency [50] and pediatric care [51, 52].

Disparities also extended to non‐pharmacologic pain management, with nHB and LatinX survivors utilizing these treatments less often than nHW survivors, mirroring patterns previously documented in non‐cancer settings [20, 21, 22, 53, 54]. Evidence specific to cancer populations remains limited. One study reported that Black cancer residents in nursing homes were less likely to receive non‐pharmacologic therapies compared to White residents, though these findings may not generalize to outpatient or hospital‐based care [55]. Beyond this, most prior work has focused on non‐cancer conditions, leaving a critical gap in understanding non‐pharmacologic pain management patterns in oncology. Our study provides some of the earliest evidence of disparities in non‐pharmacologic pain management among cancer survivors, highlighting a pressing need for additional research to explore social, cultural, and structural factors influencing utilization, and the potential role of policy and health system interventions in promoting equitable pain management.

Further, our results showed that minority survivors have lower dispensed opioid doses compared to nHW survivors, with API survivors receiving the lowest doses, despite generally longer supply durations. Similar patterns were observed in studies of gastrointestinal cancer survivors nearing end‐of‐life [45] and in Medicare claims data [56], as well as in chronic non‐cancer pain settings [46], suggesting possible clinician biases [39, 57, 58, 59, 60] including misconceptions that Black survivors feel less pain or are more prone to opioid misuse [16, 58]. However, comparisons across studies are limited due to differences in study populations and care settings. Evidence on opioid supply duration is also mixed; while some studies reported shorter supply durations for Black survivors in non‐cancer setting [61], others reported longer‐term prescriptions for Black breast cancer survivors [40]. These variations highlight the need for meta‐analysis to better understand disparities in opioid use for pain management. Interestingly, for non‐opioid analgesics, we found the reverse pattern—minority survivors received higher dispensed doses but for shorter durations. Given limited research on non‐opioid pain management in cancer care, our findings address a critical gap and add valuable insight.

The observed disparities appear to rise from interacting patient‐, provider‐, and system‐level mechanisms. At the patient level, communication barriers, mistrust in providers, concerns about addiction, or fatalistic beliefs may limit accurate pain reporting or treatment uptake among marginalized groups—patterns often shaped by historical and ongoing experiences of discrimination within healthcare. At the provider level, implicit bias and racialized stereotypes, such as assumptions that Black patients exaggerate pain or are at higher risk for opioid misuse, can influence clinical judgment and prescribing decisions, resulting in undertreatment. These dynamics are further reinforced by structural factors, including heightened regulatory scrutiny and stigmatizing narratives surrounding opioid use, which may disproportionately constrain pain management for minoritized patients [5, 62, 63, 64, 65, 66].

Our study has several strengths, including its large nationally representative cohort and comprehensive assessment of both opioid and non‐opioid pain management. To our knowledge, this is the first study to document racial‐ethnic disparities in non‐pharmacologic pain treatment among cancer survivors. However, our findings should be interpreted with certain limitations. We analyzed only filled prescriptions from claims, which may reflect access and adherence issues. This approach also does not account for over‐the‐counter medications or complementary therapies, limiting visibility into the full spectrum of pain management strategies survivors may use. We could not measure pain levels, limiting the ability to determine whether observed differences reflect patient preference or appropriate tailoring of care. The 365‐day follow‐up period after cancer diagnosis may also miss longer‐term patterns of pain management and the evolution of disparities over time. We used radiation and chemotherapy data in our analysis, obtained from SEER cancer file, which may be incomplete. Some patients may have used pain therapies for non‐cancer‐related conditions; although our new‐user study design helps reduce the influence of prior use, claims data do not include information on reasons for prescribing, which remains a general limitation. Finally, the Medicare‐only sample limits generalizability to younger survivors or those with other insurance. Future research should incorporate multiple data sources, including patient‐reported outcomes, unfilled prescriptions, and broader insurance populations, to provide a more comprehensive understanding of inequities in pain management.

## 5 Conclusion

The study highlights significant racial‐ethnic disparities in the use of prescribed pain management treatments among older cancer survivors. Both opioid and non‐opioid analgesics were utilized less frequently by minority groups. Notably, they received lower opioid doses despite longer supply durations, and higher non‐opioid doses with shorter supply durations. Disparities in non‐pharmacologic treatment utilization were particularly pronounced among nHB and Latinx survivors. These findings underscore the urgent need for targeted, equitable interventions to address persistent gaps in the utilization of cancer pain management services.

## Author Contributions

**Oindrila Bhattacharyya:** methodology, data curation, writing – original draft, writing – review and editing, visualization, project administration. **Mohamed I. Elsaid:** methodology, software, data curation, formal analysis, writing – review and editing. **Brittany E. Punches:** methodology, writing – review and editing. **Andy Ni:** methodology, writing – review and editing. **Ashley S. Felix:** conceptualization, supervision. **Macarius M. Donneyong:** conceptualization, methodology, data curation, resources, writing – original draft, writing – review and editing, supervision, funding acquisition. All authors are review and approval of the final manuscript.

## Funding

This work was supported by CCTS Pilot Grant on Secondary Analysis of Existing Datasets, OSU (UL1TR002733), the California Department of Public Health pursuant to California Health and Safety Code Section 103885; Centers for Disease Control and Prevention's (CDC) National Program of Cancer Registries, under cooperative agreement 1NU58DP007156; the National Cancer Institute's Surveillance, Epidemiology and End Results Program under contract HHSN261201800032I awarded to the University of California, San Francisco, contract HHSN261201800015I awarded to the University of Southern California, and contract HSN261201800009I awarded to the Public Health Institute.

## Conflicts of Interest

The authors declare no conflicts of interest.

## Supporting information


**Data S1:** cam471536‐sup‐0001‐TableS1‐S7.docx.

## Data Availability

The data underlying this article were obtained from the SEER‐Medicare database under a data use agreement and are not publicly available. Access to these data requires approval from the National Cancer Institute and the Centers for Medicare & Medicaid Services. Data may be shared upon reasonable request to the corresponding author, subject to approval by SEER‐Medicare.
