# Supplementary material for: Racial and Ethnic Disparities in Pharmacologic and Non‐Pharmacologic Pain Management Among Older Cancer Survivors
Source: Cancer Med. 2026 Jan 23;15(1):e71536. doi: 10.1002/cam4.71536 (PMC12828668; doi:10.1002/cam4.71536)
Supplement: Supplementary file 1 — Data S1: cam471536‐sup‐0001‐TableS1‐S7.docx. [file CAM4-15-e71536-s001.docx]

**LIST OF SUPPLEMENTAL TABLES**

Table S1: List of ICD-9 and ICD-10 codes used to identify cancer cases

Table S2: List of CPT codes to identify non-pharmacologic interventions for pain among cancer survivors

Table S3: Pain Management Use (Crude Rates) by Race-Ethnicity and Gender

Table S4: Incidence, Dose, and Supply Days (Crude Rates) of Opioid Prescriptions by Race-Ethnicity and Gender Within First 90 Days of Diagnosis

Table S5: Incidence, Dose, and Supply Days (Crude Rates) of Non-Opioid Prescriptions by Race-Ethnicity and Gender Within First 90 Days of Diagnosis

Table S6: Pain Management Use by Race-Ethnicity and Cancer Stage (N=300,048)

Table S7: Pain Management Use by Race-Ethnicity and Type of Cancer (N=300,048)

Table S1: List of ICD-9 and ICD-10 codes used to identify cancer cases

| **Cancer types** | **ICD-9-CM Codes** | **ICD-10-CM Codes** |
| --- | --- | --- |
| Breast Cancer | 174.0, 174.1, 174.2, 174.3, 174.4, 174.5, 174.6, 174.8, 174.9, 175.0, 175.9 | C50.0, C50.01, C50.011, C50.012, C50.019, C50.02, C50.021, C50.022, C50.029, C50.1, C50.11, C50.111, C50.112, C50.119, C50.12, C50.121, C50.122, C50.129, C50.2, C50.21, C50.211, C50.212, C50.219, C50.22, C50.221, C50.222, C50.229, C50.3, C50.31, C50.311, C50.312, C50.319, C50.32, C50.321, C50.322, C50.329, C50.4, C50.41, C50.411, C50.412, C50.419, C50.42, C50.421, C50.422, C50.429, C50.5, C50.51, C50.511, C50.512, C50.519, C50.52, C50.521, C50.522, C50.529, C50.6, C50.61, C50.611, C50.612, C50.619, C50.62, C50.621, C50.622, C50.629, C50.8, C50.81, C50.811, C50.812, C50.819, C50.82, C50.821, C50.822, C50.829, C50.9, C50.91, C50.911, C50.912, C50.919, C50.92, C50.921, C50.922, C50.929 |
| Lung Cancer | 162.0, 162.2, 162.3, 162.4, 162.5, 162.8, 162.9 | C34.0, C34.00, C34.01, C34.02, C34.1, C34.10, C34.11, C34.12, C34.2, C34.3, C34.30, C34.31, C34.32, C34.8, C34.80, C34.81, C34.82, C34.9, C34.90, C34.91, C34.92 |
| Colorectal Cancer | 153.0, 153.1, 153.2, 153.3, 153.4, 153.5, 153.6, 153.7, 153.8, 153.9, 154.0, 154.1, 154.2, 154.3, 154.8 | C18.0, C18.1, C18.2, C18.3, C18.4, C18.5, C18.6, C18.7, C18.8, C18.9, C19, C20 |
| Prostate Cancer | 185 | C61 |
| Uterine Cancer | 182.1, 182.8 | C55 |
| Head & Neck Cancer | 140.0, 140.1, 140.3, 140.4, 140.5, 140.6, 140.8, 140.9, 142.0, 142.1, 142.2, 142.8, 142.9, 143.0, 143.1, 143.8, 143.9, 144.0, 144.1, 144.8, 144.9, 146.0, 146.1, 146.2, 146.3, 146.4, 146.5, 146.6, 146.7, 146.8, 146.9, 147.0, 147.1, 147.2, 147.3, 147.8, 147.9, 148.0, 148.1, 148.2, 148.3, 148.8, 148.9, 149.0, 149.1, 149.8, 149.9 | C00.0. C00.1, C00.2, C00.3, C00.4, C00.5, C00.6, C00.8, C00.9, C01, C02.0, C02.1, C02.2, C02.3, C02.4, C02.8, C02.9, C03.0, C03.1, C03.9, C04.0, C04.1, C04.8, C04.9, C05.0, C05.1, C05.2, C05.8, C05.9, C06.0, C06.1, C06.2, C06.8, C06.80, C06.89, C06.9, C07, C08.0, C08.1, C08.9, C09.0, C09.1, C09.8, C09.9, C10.0, C10.1, C10.2, C10.3, C10.4, C10.8, C10.9, C11.0, C11.1, C11.2, C11.3, C11.8, C11.9, C12, C13.0, C13.1, C13.2, C13.8, C13.9, C14.0, C14.2, C14.8, C30.0, C32.0, C32.1, C32.2, C32.3, C32.8, C32.9, C31.0, C31.1, C31.2, C31.3, C31.8, C31.9 |

Table S2: List of CPT codes to identify non-pharmacologic interventions for pain among cancer survivors

| **Discipline** | **Interventions** | **CPT Codes** | **Interventions** | **CPT Codes** |
| --- | --- | --- | --- | --- |
| Physical Medicine and Rehabilitation | **Physical therapy** |  | **Recreational therapy** |  |
|  | PT evaluation | 97001 | Sensory integration | 97533 |
|  | PT re-evaluation | 97002 | Wheelchair management training | 97542 |
|  | Ultrasound therapy | 97035 | Work hardening | 97545 |
|  | Hydrotherapy | 97036 | Orthotic management and training | 97760 |
|  | Physical therapy treatment | 97039 | C/o for orthotic/prosth use | 97762 |
|  | Therapeutic exercises | 97110 |  |  |
|  | Neuromuscular reeducation | 97112 |  |  |
|  | Aquatic therapy/exercises | 97113 |  |  |
|  | Gait training therapy | 97116 |  |  |
|  | Physical medicine procedure | 97139 |  |  |
|  | Manual therapy 1/> regions | 97140 |  |  |
|  | Group therapeutic procedures | 97150 |  |  |
|  | Therapeutic activities | 97530 |  |  |
|  | **Occupational therapy** |  |  |  |
|  | OT evaluation | 97003 |  |  |
|  | Ot re-evaluation | 97004 |  |  |
|  | Self-care management training | 97535 |  |  |
|  | Community/work reintegration | 97537 |  |  |
| Integrative Therapies | Massage | 97124 |  |  |
|  | **Acupuncture** |  |  |  |
|  | Acu, initial w/out elect stim | 97810 |  |  |
|  | Acu, w/out elect stim | 97811 |  |  |
|  | Acu, initial w elect stim | 97813 |  |  |
|  | Acu, w elect stim | 97814 |  |  |

Table S2: Cont’d

| **Discipline** | **Interventions** | **CPT Codes** | **Interventions** | **CPT Codes** |
| --- | --- | --- | --- | --- |
| Interventional Therapies | **Nerve blocks** |  | N block stellate ganglion | 64510 |
|  | Anesth nerve block/inj | 01991 | N block inj hypogas plxs | 64517 |
|  | Anesth n block/inj prone | 01992 | N block lumbar/thoracic | 64520 |
|  | N block inj trigeminal | 64400 | N block inj celiac pelus | 64530 |
|  | N block inj facial | 64402 | **Neuraxial infusion (epidural/intrathecal)** |  |
|  | N block inj occipital | 64405 | Inject spine cerv/thoracic | 62310 |
|  | N block inj vagus | 64408 | Inject spine lumbar/sacral | 62311 |
|  | N block inj phrenic | 64410 | Inject spine w/cath crv/thrc | 62318 |
|  | N block inj spinal accessor | 64412 | Inject spine w/cath lmb/scrl | 62319 |
|  | N block inj cervical plexus | 64413 | Hosp manage cont drug admin | 01996 |
|  | N block inj brachial plexus | 64415 | **Vertebroplasty** |  |
|  | N block cont infuse b plex | 64416 | Percut vertebroplasty thor | 22520 |
|  | N block inj axillary | 64417 | Percut vertebroplasty lumb | 22521 |
|  | N block inj suprascapular | 64418 | Percut vertebroplasty addl | 22522 |
|  | N block inj intercost sng | 64420 | **Kyphoplasty** |  |
|  | N block inj intercost mlt | 64421 | Percut kyphoplasty thor | 22523 |
|  | N block inj ilio-ing/hypogi | 64425 | Percut kyphoplasty lumbar | 22524 |
|  | N block inj pudendal | 64430 | Percut kyphoplasty add-on | 22525 |
|  | N block inj paracervical | 64435 |  |  |
|  | N block inj sciatic sng | 64445 |  |  |
|  | N blk inj sciatic cont inf | 64446 |  |  |
|  | N block inj fem single | 64447 |  |  |
|  | N block inj fem cont inf | 64448 |  |  |
|  | N block inj lumbar plexus | 64449 |  |  |
|  | N block other peripheral | 64450 |  |  |
|  | N block inj plantar digit | 64455 |  |  |
|  | Inj foramen epidural c/t | 64479 |  |  |
|  | Inj foramen epidural l/s | 64483 |  |  |
|  | Inj paravert f jnt c/t 1 lev | 64490 |  |  |
|  | Inj paravert f jnt l/s 1 lev | 64493 |  |  |
|  | N block spenopalatine gangl | 64505 |  |  |
|  | N block carotid sinus s/p | 64508 |  |  |

Table S2: Cont’d

| **Discipline** | **Interventions** | **CPT Codes** | **Interventions** | **CPT Codes** |
| --- | --- | --- | --- | --- |
| Psychological Approaches | **Cognitive behavioral therapy** |  | Periph field stimul analys | 0285T |
|  | Cognitive skills development | 97532 | **Transcranial stimulation** |  |
|  | Distraction | 97127 | Nervous system surgery | 64999 |
|  | Mindfulness | 90837 | Tcranial magn stim tx plan | 90867 |
|  | Guided imagery | 90837 | Tcranial magn stim tx deli | 90868 |
|  | TENS | 97014 | Tcran magn stim redetemine | 90869 |
|  | Periph field stimul revise | 0284T |  |  |
| Neurostimulatory Therapies | Apply neurostimulator | 64550 |  |  |
|  | Implant neuroelectrodes | 64555 |  |  |
|  | Electric stimulation therapy | 97014 |  |  |
|  | Electrical stimulation | 97032 |  |  |
|  | **Spinal cord stimulation** |  |  |  |
|  | Implant neuroelectrodes | 63650 |  |  |
|  | Implant neuroelectrodes | 63655 |  |  |
|  | Remove spine eltrd perq aray | 63661 |  |  |
|  | Remove spine eltrd plate | 63662 |  |  |
|  | Revise spine eltrd perq aray | 63663 |  |  |
|  | Revise spine eltrd plate | 63664 |  |  |
|  | Insrt/redo spine n generator | 63685 |  |  |
|  | Revise/remove neuroreceiver | 63688 |  |  |
|  | **Peripheral nerve stimulation** |  |  |  |
|  | Insrt/redo neurostim 1 array | 61885 |  |  |
|  | Apply neurostimulator | 64550 |  |  |
|  | Implant neuroelectrodes | 64553 |  |  |
|  | Implant neuroelectrodes | 64555 |  |  |
|  | Implant neuroelectrodes | 64561 |  |  |
|  | Revise/repl vagus n eltrd | 64569 |  |  |
|  | Remove vagus n eltrd | 64570 |  |  |
|  | Implant neuroelectrodes | 64575 |  |  |
|  | Implant neuroelectrodes | 64581 |  |  |
|  | Revise/remove neuroelectrode | 64585 |  |  |
|  | Insrt/redo pn/gastr stimul | 64590 |  |  |
|  | Revise/rmv pn/gastr stimul | 64595 |  |  |
|  | Periph field stimul trial | 0282T |  |  |
|  | Periph field stimul perm | 0283T |  |  |

Table S3: Pain Management Use (Crude Rates) by Race-Ethnicity and Gender

| **Race-Ethnicity**  **and Gender** | **Any Pain**  **Management (95% CI)** (N=300,048) | | **Pharmacologic Pain**  **Management (95% CI)** (n=175,336) | | **Non-Pharmacologic Pain**  **Management (95% CI)** (n=124,712) | |
| --- | --- | --- | --- | --- | --- | --- |
|  | **Crude Incidence (%)** | **Crude**  **Incidence Ratio** | **Crude Incidence (%)** | **Crude**  **Incidence Ratio** | **Crude Incidence (%)** | **Crude**  **Incidence Ratio** |
| **All** |  |  |  |  |  |  |
| nHW | 54.8 (54.6-55.0) | Ref. | 52.2 (52.0-52.4) | Ref. | 7.0 (6.9-7.1) | Ref. |
| nHB | 50.3 (49.8-50.9) | 0.92 (0.91-0.93) | 48.4 (47.8-49.0) | 0.93 (0.92-0.94) | 4.5 (4.2-4.7) | 0.64 (0.61-0.68) |
| LatinX | 51.6 (51.0-52.2) | 0.94 (0.93-0.95) | 49.8 (49.2-50.4) | 0.95 (0.94-0.97) | 4.7 (4.4-4.9) | 0.67 (0.63-0.71) |
| API | 52.6 (51.9-53.2) | 0.96 (0.95-0.97) | 50.1 (49.4-50.8) | 0.96 (0.95-0.97) | 6.7 (6.4-7.1) | 0.97 (0.92-1.02) |
| Other | 34.8 (33.4-36.3) | 0.64 (0.61-0.66) | 31.3 (29.9-32.7) | 0.60 (0.57-0.63) | 5.6 (4.9-6.3) | 0.81 (0.71-0.91) |
| **Male** |  |  |  |  |  |  |
| nHW | 44.3 (44.0-44.6) | Ref. | 41.1 (40.8-41.4) | Ref. | 6.8 (6.6-6.9) | Ref. |
| nHB | 38.6 (37.8-39.4) | 0.87 (0.85-0.89) | 36.2 (35.4-37.0) | 0.88 (0.86-0.90) | 4.5 (4.1-4.8) | 0.66 (0.61-0.71) |
| LatinX | 41.1 (40.3-42.0) | 0.93 (0.91-0.95) | 38.9 (38.1-39.7) | 0.95 (0.93-0.97) | 4.5 (4.2-4.8) | 0.66 (0.61-0.72) |
| API | 43.7 (42.8-44.6) | 0.99 (0.96-1.01) | 40.7 (39.8-41.6) | 0.99 (0.97-1.01) | 6.8 (6.3-7.3) | 1.00 (0.93-1.08) |
| Other | 25.7 (24.2-27.3) | 0.58 (0.55-0.62) | 21.3 (19.9-22.8) | 0.52 (0.48-0.56) | 5.9 (5.1-6.7) | 0.87 (0.75-1.00) |
| **Female** |  |  |  |  |  |  |
| nHW | 64.3 (64.0-64.6) | Ref. | 62.2 (61.9-62.5) | Ref. | 7.1 (7.0-7.3) | Ref. |
| nHB | 61.9 (61.2-62.7) | 0.96 (0.95-0.98) | 60.6 (59.8-61.4) | 0.97 (0.96-0.99) | 4.5 (4.1-4.8) | 0.63 (0.58-0.68) |
| LatinX | 64.0 (63.1-64.8) | 1.00 (0.98-1.01) | 62.5 (61.7-63.4) | 1.01 (0.99-1.02) | 4.9 (4.5-5.3) | 0.68 (0.63-0.74) |
| API | 61.5 (60.6-62.4) | 0.96 (0.94-0.97) | 59.6 (58.7-60.5) | 0.96 (0.94-0.97) | 6.7 (6.2-7.2) | 0.94 (0.87-1.01) |
| Other | 60.4 (57.6-63.4) | 0.94 (0.90-0.99) | 59.4 (56.5-62.3) | 0.95 (0.91-1.00) | 4.9 (3.8-6.4) | 0.69 (0.53-0.89) |

Note: Pharmacologic pain management includes use of either opioid or non-opioid analgesics. The models were adjusted for age at cancer diagnosis, year of diagnosis, type of cancer, cancer stage at diagnosis, radiation therapy, surgical therapy, chemotherapy, Charlson Comorbidity Index, type of comorbidities. Non-Hispanic White survivors are the reference group. CI = Confidence intervals, nHW = Non-Hispanic Whites, nHB = Non-Hispanic Blacks, LatinX = Hispanic-Latino, API = Asian/Pacific Islander.

Table S4: Incidence, Dose, and Supply Days (Crude Rates) of Opioid Prescriptions by Race-Ethnicity and Gender Within First 90 Days of Diagnosis

| **Race-Ethnicity**  **and Gender** | **Opioid Analgesics (95% CI)** (n=139,598) | | | |
| --- | --- | --- | --- | --- |
|  | **Crude Incidence (%)** | **Crude Incidence Ratio** | **Dose (MME/day), Crude Difference** | **Supply Days, Crude Difference** |
| **All** |  |  |  |  |
| nHW | 47.5 (47.3-47.7) | Ref. | Ref. | Ref. |
| nHB | 44.3 (43.7-44.9) | 0.93 (0.92-0.95) | -3.6 (-4.1 to -3.1) | 0.80 (0.67 To 0.93) |
| LatinX | 45.3 (44.7-45.9) | 0.95 (0.94-0.97) | -4.6 (-5.1 to -4.1) | 0.73 (0.60 To 0.86) |
| API | 44.8 (44.2-45.5) | 0.94 (0.93-0.96) | -6.1 (-6.7 to -5.6) | 0.31 (0.19 To 0.44) |
| Other | 27.5 (26.1-28.8) | 0.58 (0.55-0.61) | -3.9 (-5.4 to -2.3) | 0.38 (-0.05 To 0.82) |
| **Male** |  |  |  |  |
| nHW | 36.6 (36.3-36.9) | Ref. | Ref. | Ref. |
| nHB | 32.4 (31.7-33.2) | 0.89 (0.87-0.91) | -3.9 (-4.7 to -3.0) | 0.77 (0.55 To 0.99) |
| LatinX | 34.6 (33.8-35.3) | 0.95 (0.92-0.97) | -5.2 (-6.0 to -4.4) | 0.80 (0.58 To 1.01) |
| API | 35.2 (34.3-36.1) | 0.96 (0.94-0.99) | -5.7 (-6.6 to -4.8) | 0.47 (0.24 To 0.70) |
| Other | 17.6 (16.4-19.0) | 0.48 (0.45-0.52) | -4.6 (-6.9 to -2.4) | 0.47 (-0.25 To 1.18) |
| **Female** |  |  |  |  |
| nHW | 57.4 (57.1-57.7) | Ref. | Ref. | Ref. |
| nHB | 56.0 (55.2-56.8) | 0.98 (0.96-0.99) | -3.5 (-4.2 to -2.8) | 0.82 (0.67 To 0.97) |
| LatinX | 57.9 (57.1-58.8) | 1.01 (0.99-1.03) | -4.1 (-4.8 to -3.4) | 0.62 (0.47 To 0.78) |
| API | 54.6 (53.7-55.5) | 0.95 (0.93-0.97) | -6.4 (-7.1 to -5.7) | 0.18 (0.03 To 0.33) |
| Other | 55.2 (52.3-58.2) | 0.96 (0.91-1.01) | -3.0 (-5.1 to -0.85) | 0.16 (-0.36 To 0.68) |

Note: Dose was measured as first opioid dose filled. The models were adjusted for age at cancer diagnosis, year of diagnosis, type of cancer, cancer stage at diagnosis, radiation therapy, surgical therapy, chemotherapy, Charlson Comorbidity Index, type of comorbidities. MME = morphine milligram equivalents per day of the first prescription filled. CI = Confidence intervals, nHW = Non-Hispanic Whites, nHB = Non-Hispanic Blacks, LatinX = Hispanic-Latino, API = Asian/Pacific Islander.

Table S5: Incidence, Dose, and Supply Days (Crude Rates) of Non-Opioid Prescriptions by Race-Ethnicity and Gender Within First 90 Days of Diagnosis

| **Race-Ethnicity**  **and Gender** | **Non-Opioid Analgesics (95% CI)** (n=35,738) | | | |
| --- | --- | --- | --- | --- |
|  | **Crude Incidence (%)** | **Crude Incidence Ratio** | **Dose (mg), Crude Difference** | **Supply Days, Crude Difference** |
| **All** |  |  |  |  |
| nHW | 12.3 (12.1-12.4) | Ref. | Ref. | Ref. |
| nHB | 10.8 (10.5-11.2) | 0.88 (0.85-0.92) | 56.2 (33.5-73.5) | -2.13 (-2.91 To -1.35) |
| LatinX | 10.9 (10.5-11.3) | 0.89 (0.86-0.92) | 87.2 (63.2-103.4) | 1.22 (0.31 To 2.14) |
| API | 12.0 (11.6-12.5) | 0.98 (0.94-1.02) | -17.0 (-35.7-1.8) | -0.55 (-1.44 To 0.34) |
| Other | 7.2 (6.5-8.1) | 0.59 (0.53-0.66) | 131.4 (55.3-207.5) | 3.03 (0.13 To 5.92) |
| **Male** |  |  |  |  |
| nHW | 10.0 (9.8-10.2) | Ref. | Ref. | Ref. |
| nHB | 8.3 (7.8-8.7) | 0.83 (0.78-0.88) | 34.0 (-1.1 to 69.2) | -0.89 (-2.20 To 0.41) |
| LatinX | 8.9 (8.4-9.4) | 0.89 (0.84-0.94) | 84.1 (47.2-121.0) | 1.76 (0.43 To 3.10) |
| API | 11.0 (10.4-11.6) | 1.10 (1.04-1.16) | -31.9 (-59.3 to -4.5) | -0.61 (-1.90 To 0.68) |
| Other | 5.8 (5.1-6.7) | 0.58 (0.51-0.67) | 85.2 (-4.8 to 175.2) | 6.88 (2.82 To 10.94) |
| **Female** |  |  |  |  |
| nHW | 14.3 (14.1-14.5) | Ref. | Ref. | Ref. |
| nHB | 13.4 (12.9-14.0) | 0.94 (0.90-0.98) | 69.5 (40.2-98.9) | -2.86 (-3.84 To -1.89) |
| LatinX | 13.2 (12.6-13.8) | 0.92 (0.88-0.97) | 85.1 (53.4 to 116.8) | 0.87 (-0.38 To 2.11) |
| API | 13.1 (12.4-13.7) | 0.91 (0.87-0.96) | -11.8 (-37.5 to 14.0) | -0.43 (-1.65 To 0.79) |
| Other | 11.3 (9.5-13.3) | 0.79 (0.67-0.93) | 171.4 (39.7 to 303.1) | -2.02 (-5.86 To 1.81) |

Note: Dose was measured as first opioid dose filled. The models were adjusted for age at cancer diagnosis, year of diagnosis, type of cancer, cancer stage at diagnosis, radiation therapy, surgical therapy, chemotherapy, Charlson Comorbidity Index, type of comorbidities. MME = morphine milligram equivalents per day of the first prescription filled. CI = Confidence intervals, nHW = Non-Hispanic Whites, nHB = Non-Hispanic Blacks, LatinX = Hispanic-Latino, API = Asian/Pacific Islander.

Table S6: Pain Management Use by Race-Ethnicity and Cancer Stage (N=300,048)

| **Race-Ethnicity** | **Any Pain Management**  **Adjusted^†^ Incidence Ratio** (95% CI) | **Pharmacologic Pain Management**  **Adjusted^†^ Incidence Ratio** (95% CI) | **Non-Pharmacologic Pain Management**  **Adjusted^†^ Incidence Ratio** (95% CI) |
| --- | --- | --- | --- |
| **Stage 0** |  |  |  |
| nHW | Ref | Ref | Ref |
| nHB | 0.96 (0.92 - 1.00) | 0.98 (0.94 to 1.02) | 0.58 (0.45 - 0.75) |
| LatinX | 0.98 (0.94 - 1.03) | 1.00 (0.95 to 1.05) | 0.55 (0.41 - 0.74) |
| API | 0.87 (0.83 - 0.92) | 0.89 (0.84 to 0.93) | 0.65 (0.50 - 0.86) |
| Other | 0.93 (0.81 - 1.07) | 0.92 (0.80 to 1.07) | 1.31 (0.76 - 2.27) |
| **Stage I** |  |  |  |
| nHW | Ref | Ref | Ref |
| nHB | 0.97 (0.95 - 0.99) | 0.99 (0.97 to 1.02) | 0.60 (0.53 - 0.67) |
| LatinX | 0.99 (0.97 - 1.01) | 1.00 (0.98 to 1.02) | 0.65 (0.58 - 0.73) |
| API | 0.97 (0.95 - 1.00) | 0.98 (0.95 to 1.00) | 0.94 (0.86 - 1.04) |
| Other | 0.97 (0.91 - 1.04) | 1.00 (0.93 to 1.07) | 0.56 (0.37 - 0.84) |
| **Stage II** |  |  |  |
| nHW | Ref | Ref | Ref |
| nHB | 0.97 (0.95 - 0.99) | 0.99 (0.97 to 1.01) | 0.70 (0.64 - 0.76) |
| LatinX | 0.98 (0.96 - 1.00) | 1.01 (0.99 to 1.03) | 0.66 (0.60 - 0.73) |
| API | 0.96 (0.93 - 0.98) | 0.95 (0.93 to 0.98) | 0.98 (0.90 - 1.07) |
| Other | 0.82 (0.77 - 0.88) | 0.76 (0.7 to 0.82) | 1.14 (0.95 - 1.37) |
| **Stage III** |  |  |  |
| nHW | Ref | Ref | Ref |
| nHB | 0.97 (0.94 - 0.99) | 0.97 (0.95 to 1.00) | 0.60 (0.51 - 0.69) |
| LatinX | 0.97 (0.95 - 1.00) | 0.98 (0.95 to 1.01) | 0.75 (0.65 - 0.86) |
| API | 1.00 (0.97 - 1.02) | 0.99 (0.96 to 1.02) | 1.04 (0.92 - 1.18) |
| Other | 1.05 (0.94 - 1.17) | 1.06 (0.95 to 1.18) | 0.98 (0.60 - 1.59) |
| **Stage IV** |  |  |  |
| nHW | Ref | Ref | Ref |
| nHB | 0.97 (0.95 - 1.00) | 0.98 (0.95 to 1.00) | 0.70 (0.59 - 0.83) |
| LatinX | 1.02 (0.99 - 1.05) | 1.03 (1.00 to 1.06) | 0.77 (0.63 - 0.93) |
| API | 0.99 (0.96 - 1.02) | 0.98 (0.95 to 1.01) | 1.35 (1.17 - 1.56) |
| Other | 1.02 (0.91 - 1.15) | 1.04 (0.93 to 1.17) | 0.90 (0.43 - 1.86) |

Note: Pharmacologic pain management includes use of either opioid or non-opioid analgesics. The models were adjusted for age at cancer diagnosis, year of diagnosis, type of cancer, cancer stage at diagnosis, radiation therapy, surgical therapy, chemotherapy, Charlson Comorbidity Index, type of comorbidities. CI = Confidence intervals, nHW = Non-Hispanic Whites, nHB = Non-Hispanic Blacks, LatinX = Hispanic-Latino, API = Asian/Pacific Islander.

Table S7: Pain Management Use by Race-Ethnicity and Type of Cancer (N=300,048)

| **Race-Ethnicity** | **Any Pain Management**  **Adjusted Incidence Ratio** (95% CI) | **Pharmacologic Pain Management**  **Adjusted^†^ Incidence Ratio** (95% CI) | **Non-Pharmacologic Pain Management**  **Adjusted Incidence Ratio** (95% CI) |
| --- | --- | --- | --- |
| **Prostate** |  |  |  |
| nHW | Ref | Ref | Ref |
| nHB | 0.94 (0.91 - 0.97) | 0.96 (0.93 - 0.99) | 0.75 (0.68 - 0.83) |
| LatinX | 0.96 (0.93 - 0.99) | 1.00 (0.96 - 1.03) | 0.70 (0.63 - 0.77) |
| API | 0.92 (0.89 - 0.96) | 0.92 (0.88 - 0.96) | 0.96 (0.86 - 1.07) |
| Other | 0.80 (0.75 - 0.86) | 0.71 (0.66 - 0.78) | 1.23 (1.06 - 1.44) |
| **Breast** |  |  |  |
| nHW | Ref | Ref | Ref |
| nHB | 0.95 (0.93 - 0.96) | 0.95 (0.94 - 0.97) | 0.54 (0.48 - 0.61) |
| LatinX | 0.96 (0.95 - 0.98) | 0.97 (0.95 - 0.99) | 0.62 (0.54 - 0.70) |
| API | 0.94 (0.92 - 0.96) | 0.94 (0.92 - 0.96) | 0.83 (0.74 - 0.93) |
| Other | 0.98 (0.92 - 1.04) | 0.98 (0.93 - 1.04) | 0.69 (0.46 - 1.01) |
| **Lung** |  |  |  |
| nHW | Ref | Ref | Ref |
| nHB | 0.97 (0.94 - 0.99) | 0.98 (0.95 - 1.00) | 0.62 (0.54 - 0.70) |
| LatinX | 1.02 (0.99 - 1.05) | 1.03 (1.00 - 1.06) | 0.76 (0.67 - 0.86) |
| API | 0.98 (0.95 - 1.00) | 0.98 (0.95 - 1.00) | 1.09 (0.99 - 1.20) |
| Other | 1.00 (0.90 - 1.11) | 1.03 (0.93 - 1.15) | 0.57 (0.32 - 1.01) |
| **Colorectal** |  |  |  |
| nHW | Ref | Ref | Ref |
| nHB | 1.01 (0.98 - 1.03) | 1.04 (1.01 - 1.07) | 0.62 (0.55 - 0.70) |
| LatinX | 1.02 (0.99 - 1.04) | 1.04 (1.01 - 1.07) | 0.71 (0.63 - 0.80) |
| API | 0.99 (0.97 - 1.02) | 0.99 (0.96 - 1.02) | 1.08 (0.98 - 1.20) |
| Other | 0.99 (0.91 - 1.09) | 1.01 (0.91 - 1.11) | 1.21 (0.88 - 1.66) |
| **Uterine** |  |  |  |
| nHW | Ref | Ref | Ref |
| nHB | 0.99 (0.96 - 1.03) | 1.01 (0.97 - 1.05) | 0.83 (0.68 - 1.02) |
| LatinX | 1.00 (0.97 - 1.04) | 1.01 (0.97 - 1.05) | 0.68 (0.54 - 0.85) |
| API | 0.99 (0.95 - 1.04) | 0.99 (0.95 - 1.04) | 0.82 (0.64 - 1.05) |
| Other | 1.06 (0.94 - 1.21) | 1.09 (0.95 - 1.24) | 0.62 (0.26 - 1.47) |
| **Head and Neck** |  |  |  |
| nHW | Ref | Ref | Ref |
| nHB | 0.97 (0.91 - 1.04) | 0.98 (0.92 - 1.04) | 0.87 (0.52 - 1.45) |
| LatinX | 1.00 (0.95 - 1.05) | 1.00 (0.95 - 1.06) | 0.85 (0.55 - 1.31) |
| API | 1.05 (0.99 - 1.10) | 1.04 (0.98 - 1.10) | 1.53 (1.09 - 2.15) |
| Other | 0.87 (0.73 - 1.04) | 0.88 (0.73 - 1.05) | 0.57 (0.14 - 2.26) |

Note: Pharmacologic pain management includes use of either opioid or non-opioid analgesics. The models were adjusted for age at cancer diagnosis, year of diagnosis, type of cancer, cancer stage at diagnosis, radiation therapy, surgical therapy, chemotherapy, Charlson Comorbidity Index, type of comorbidities. CI = Confidence intervals, nHW = Non-Hispanic Whites, nHB = Non-Hispanic Blacks, LatinX = Hispanic-Latino, API = Asian/Pacific Islander.
